# Assessments of different inactivating reagents in formulating transmissible gastroenteritis virus vaccine

**DOI:** 10.1186/s12985-020-01433-8

**Published:** 2020-10-23

**Authors:** Fujie Zhao, Lintao Liu, Menglong Xu, Xiangli Shu, Lanlan Zheng, Zhanyong Wei

**Affiliations:** 1 The College of Veterinary Medicine, Henan Agricultural University, Nongye Road 63#, Zhengzhou, 450002 Henan Province People’s Republic of China; 2 Key Laboratory for Animal-Derived Food Safety of Henan Province, Zhengzhou, 450002 Henan China

**Keywords:** Transmissible gastroenteritis virus (TGEV), Formaldehyde, β-propiolactone, Binary-ethylenimine, Inactivated vaccine

## Abstract

**Background:**

Transmissible gastroenteritis virus (TGEV) causes enteric infection in piglets, characterized by vomiting, severe diarrhea and dehydration, and the mortality in suckling piglets is often high up to 100%. Vaccination is an effective measure to control the disease caused by TGEV.

**Methods:**

In this study, cell-cultured TGEV HN-2012 strain was inactivated by formaldehyde (FA), β-propiolactone (BPL) or binaryethylenimine (BEI), respectively. Then the inactivated TGEV vaccine was prepared with freund's adjuvant, and the immunization effects were evaluated in mice. The TGEV-specific IgG level was detected by ELISA. The positive rates of CD4^+^, CD8^+^, CD4^+^IFN-γ^+^, CD4^+^IL-4^+^ T lymphocytes were detected by flow cytometry assay. Lymphocyte proliferation assay and gross pathology and histopathology examination were also performed to assess the three different inactivating reagents in formulating TGEV vaccine.

**Results:**

The results showed that the TGEV-specific IgG level in FA group (n = 17) was earlier and stronger, while the BEI group produced much longer-term IgG level. The lymphocyte proliferation test demonstrated that the BEI group had a stronger ability to induce spleen lymphocyte proliferation. The positive rates of CD4^+^ and CD8^+^ T lymphocyte subsets of peripheral blood lymphocyte in BEI group was higher than that in FA group and BPL groups by flow cytometry assay. The positive rate of CD4^+^IFN-γ^+^ T lymphocyte subset was the highest in the BPL group, and the positive rate of CD4^+^IL-4^+^ T lymphocyte subset was the highest in the FA group. There were no obvious pathological changes in the vaccinated mice and the control group after the macroscopic and histopathological examination.

**Conclusions:**

These results indicated that all the three experimental groups could induce cellular and humoral immunity, and the FA group had the best humoral immunity effect, while the BEI group showed its excellent cellular immunity effect.

## Introduction

Transmissible gastroenteritis virus (TGEV) is an enveloped, positive, single-stranded RNA virus, which belongs to the *Alphacoronavirus* genus, *Coronaviridae* family. TGEV causes acute enteric disease in pigs, characterized by vomiting, severe diarrhea and dehydration. The mortality of TGEV often reaches 100% in suckling piglets less than two weeks of age, and causes huge economic losses in pig industry around the world [1, 2]. Until now, there is no effective drug to treat TGEV infection, and vaccination should be the effective measure to control the disease caused by TGEV [3, 4].

To prevent TGEV infection, several vaccine technologies have been developed, including inactivated vaccine, attenuated vaccine, DNA vaccine, recombinant vaccine, vectored vaccine, and even multiple vaccines which are often combined with rotavirus and porcine epidemic diarrhea virus (PEDV) [5, 6]. For inactivated vaccines, viruses are completely inactivated by chemicals with an excellent safety, as well as good tolerance and few adverse reactions [7]. Thus, virus inactivation is a crucial step in production of vaccines, which need to inhibit virus replication without altering their antigenicity.

The commercial inactivated TGEV vaccines were mainly inactivated by formaldehyde (FA), which is a traditional inactivating agent that frequently used in many vaccines. FA mainly focuses on viral proteins [8], and results in the alkylation of amino and affects the fusion ability of viruses. β-propiolactone (BPL) is another inactivating agent that mainly attacks nucleic acids, thus would not change the antigenic component of viruses [9]. In addition, some studies showed that BPL could also affect viral proteins [10]. Comparing the effects of two inactivating agents on Newcastle disease virus (NDV) and influenza virus, the results indicated that the potencies of FA and BPL inactivated vaccines were different [11, 12]. In case of binary-ethylenimine (BEI), its inactivation mechanism is similar to BPL and reacts very little with viral proteins. However, some studies showed that BEI could better preserve the conformation and accessibility of viral epitopes than FA and BPL [13–15].

In our study, three different inactivating agents (FA, BPL, BEI) were used to inactivate the TGEV HN-2012 strain. The immunogenicity of the inactivated TGEV vaccines in mice was evaluated, and these data may provide an assessment of different inactivating agents on vaccines.

## Materials and methods

### Cells and virus

Swine testis (ST) cells were maintained in Dulbecco's modified eagle medium (DMEM, Gibco) with 10% fetal bovine serum (FBS, Gibco) and cultured in a CO_2_ incubator at 37 °C for serially passaging and propagating of TGEV. The monolayers of ST cells were maintained in DMEM with 0% FBS, and TGEV was propagated by inoculating with 0.1 multiplicity of infection (MOI). The TGEV used in our research was the passage 15 (P15) of TGEV HN-2012 strain isolated and identified in our laboratory. The propagated TGEV culture was harvested when the cytopathic effect (CPE) was > 80%, and the virus titer was determined as 10^8.0^ TCID_50_/0.1 mL. The cell debris was removed by 8 000 rpm centrifugation for 30 min at 4 °C. TGEV was then purified with ultra-centrifugation and sucrose density gradient-centrifugation by 30 000 rpm for 3 h at 4 °C, respectively. The purified TGEV was then diluted with phosphate buffer saline (PBS) and filtered by 0.2 µm pore size filter.

### Inactivation protocols of TGEV

Three inactivating agents were selected (FA, BPL and BEI) in our study. For FA inactivation agent, 40% FA (Sinopharm Chemical Reagent Co, Ltd) was used at a final concentration of 0.1%, 0.2%, 0.3% (v/v) respectively. Different concentrations of FA were incubated with TGEV at 37 °C and collected after 6, 12, 18, 24, 30 and 36 h, respectively. The reactions were terminated by adding the sterilized 1 M sodium thiosulfate (Sinopharm Chemical Reagent Co., Ltd.) at the concentration 10 times of the final FA concentration. For BPL (Acros Organics, Geel, Belgium) inactivation agent, 0.01%, 0.02%, 0.03% (v/v) were used as the final concentrations respectively. These three concentrations of BPL were incubated with TGEV at 4 °C and collected after 6, 12, 18, 24, 30 and 36 h, respectively. The reactions were stopped in water bath at 37 °C for 2 h. BEI was prepared as described below. Briefly, the 2-bromo-ethylamine HBr (BEA) (Sigma-Aldrich, USA) was dissolved in 0.2 mol/L NaOH (Sinopharm Chemical Reagent Co., Ltd.) to obtain the BEI with the final concentration of 0.1 M. The solution was then incubated at 37 °C for 1 h and BEI was formed. Three concentrations of 0.03%, 0.04%, 0.05% (v/v) of BEI were incubated with TGEV at 30 °C and collected after 6, 12, 18, 24, 30 and 36 h, respectively. The reactions were stopped by adding sterilized 1 M sodium thiosulfate 10 times of the final BEI concentration. The experiments were performed 3 times.

### Tests of infectivity and sterility of TGEV

In order to determine the infectivity of TGEV after treatment with three inactivating reagents, inactivated TGEV was collected and cultured in ST cells for three passages. The CPE was observed and TCID_50_ titers were calculated. Untreated TGEV was used as the positive control, and DMEM was the negative control. Sterility tests were conducted in common nutrient agar and ordinary broth. Bacterial growth was observed on the culture medium after 30 h at 37 °C.

### Preparation of inactivated TGEV vaccine

To produce the inactivated TGEV vaccine, the inactivated viral antigens were emulsified with Freund's complete adjuvant (Sigma-Aldrich, USA) at a ratio of 1:1 (v/v), and this vaccine was used for the first immunization of mice. For the second and the third immunization, the inactivated TGEV antigen was emulsified with Freund's incomplete adjuvant (Sigma-Aldrich, USA) at a ratio of 1:1 (v/v).

### Vaccines immunization of mice

Sixty-eight female healthy 6–8-week-old BALB/c mice were purchased from Henan Province Laboratory Animal Management Committee in China for immunizing vaccines. And all mice were detected by the enzyme-linked immunosorbent assay (ELISA) kit (Wuhankeqian Animal Biological Products Co., Ltd.) to make sure the TGEV antibody was negative before injection. The mice were then randomly divided into 4 groups (n = 17/group), and housed separately. Mice were subcutaneously injected into backs with 200 μL of FA inactivated TGEV vaccine (group 1), 200 μL of BPL inactivated TGEV vaccine (group 2), 200 μL of BEI inactivated TGEV vaccine (group 3), and 200 μL PBS (group 4) as negative control. In this research, vaccine injections were performed three times at two-week intervals. Animal experiments in this study were carried out in accordance with the Health guide for the care and use of Laboratory animals of Henan Agricultural University.

### Detection of TGEV-specific IgG by indirect ELISA

Blood samples were randomly collected from 5 mice in each group at 0, 1, 2, 3, 4, 5, 6, 7 and 8 week after the initial immunization, and placed at 37 °C for 1 h. After centrifugation at 3500 rpm for 10 min, the serum from each mouse was collected. IgG was detected by TGEV antibody-IgG ELISA kit (Wuhankeqian Animal Biological Products Co., Ltd.). All the steps in the kit were followed. Briefly, 50 μL of 5 μg/mL purified TGEV antigen was coated to ELISA plates at 4 °C overnight. After blocked with PBS containing 1% BSA (w/v), the sera collected from mice were diluted with PBS and incubated on ELISA plates at 37 °C for 1 h. Then the horseradish peroxidase-conjugated goat anti-mouse IgG was added. The optical density (OD) 450 was measured. According to the instruction of the ELISA kit, the samples considered to be positive when OD_450_ values of the experimental groups were greater than or equal to 2.1 times of the values of the control group. Values < 0.05 were excluded.

### Detection of CD4^+^, CD8^+^ T lymphocytes

300 μL of blood samples were randomly collected from eyeballs of 3 mice in each group at 21 day post-inoculation (dpi) and 35 dpi of the first immunization, respectively. The positive rates of CD4^+^, CD8^+^ T lymphocyte subsets were analyzed by flow cytometry. Briefly, 3 mL of red blood cell lysis buffer (Solarbio) was added to each sample to completely lyse red blood cells. Then the samples were washed and re-suspended with DMEM to 1 × 10^6^ cells/mL. Cells were transferred to 48-well plates with 100 μL volume of each well, 2 μL of cell activation cocktail (Bio legend) and 1 μL of BrefeldinA (Bio legend) were added to each sample and incubated at 37 °C for 6 h. After washed and re-suspended with PBS, samples were incubated with specific fluorescent antibodies (Bio legend) of Brilliant Violet 510 (BV510) conjugated anti-mouse CD3 antibody (0.4 μg/sample), PerCP/Cyanine 5.5 conjugated anti-mouse CD4 antibody (0.2 μg/sample), and fluorescein isothiocyanate (FITC) conjugated anti-mouse CD8a antibody (1 μg/sample) for 30 min at room temperature in dark according to the manufacturer’s guidelines. All samples were stained in triplicate. The samples were analyzed by flow cytometry with a BD FACS Canto plus (BD Biosciences, US). Data were analyzed using Canto diva software (BD Biosciences, US).

### Detection of CD4^+^IFN-γ^+^, CD4^+^IL-4^+^ T lymphocytes

To further determine the levels of CD4^+^IFN-γ^+^, CD4^+^IL-4^+^ T lymphocyte subsets, cells treated in the previous step were fixed with 500 μL of 4% FA for 20 min in dark, ruptured with 1 mL of Permeabilization Wash Buffer (Bio legend). Then the cells were stained with allophycocyanin (APC) conjugated anti-mouse IFN-γ antibody (0.8 μg/sample), phycoerythrin (PE) conjugated anti-mouse IL-4 antibody (0.2 μg/sample) (Biolegend) for 30 min. All samples were stained in triplicate. These samples were analyzed by flow cytometry, and data were analyzed.

### Lymphocyte proliferation assay

At 14, 21 and 35 dpi, lymphocytes were isolated randomly from spleens of 3 mice in each group. The protocol of lymphocyte isolation from spleen was modified from the previous study [16]. The isolated lymphocytes were re-suspended in DMEM and adjusted to 1.0 × 10^6^/mL. Cells were cultured in 96-well flat-bottom plates with 100 μL per well, and stimulated with concanavalin A (ConA, Sigma) with the final concentration of 50 μg/mL or 20 μL of inactivated TGEV antigen (1 × 10^5^ TCID_50_/mL) in each well, respectively. DMEM was used as the negative control. All treatments were in triplicate. The plates were incubated at 37 °C for 20 h, and then added methylthiazoltetrazolium (MTT) (5 mg/mL) with 10 μL/well for further incubation at 37 °C for 4 h. 100 μL of 10% dimethyl sulfoxide (DMSO, Solarbio) was added to each well to stop the reaction. The OD_492_ value was determined. The stimulation index (SI) was calculated with the following formula: SI = (OD sample well − OD blank well)/(OD negative well − OD blank well).

### Gross pathology and histopathology

At 35 dpi, 3 mice in each group were randomly selected and killed. The major organs included heart, liver, spleen, lung, kidney, small intestines (jejunum and ileum) and back muscles of the injection site with vaccine were examined grossly, and then fixed with 10% formalin for 48 h. To evaluate whether the vaccine had adverse effects on mice, these fixed tissues were stained with Mayer’s H.E for histopathological examination.

### Statistical analysis

Data of the three experimental groups and the control group were evaluated by SPSS 17.0 software, and error bars represented standard deviations. Results were unpaired two-way analysis of variance (ANOVA). The results were expressed as mean ± standard deviation (SD), with *P *values < 0.05, *P *values < 0.01 and *P *values < 0.001 considered to be statistically high, significantly high and extremely high, respectively.

## Results

### Inactivation of TGEV with FA, BPL and BEI

In order to compare the effects of three inactivating agents, TGEV HN-2012 strain were treated with different concentrations of FA, BPL and BEI at different time, and the untreated virus was the positive control. TCID_50_ was calculated on ST cells for three passages to determine the infectivity of TGEV after inactivation. As shown in Table 1, final concentrations of 0.1%, 0.2% and 0.3% of FA inactivation agent were chosen, and TGEV could be completely inactivated after 18 h. For BPL inactivation agent, the final concentrations of 0.01% and 0.02% could inactivate TGEV at after 12 h, and the final concentration of 0.03% could inactivate TGEV at 6 h. In case of BEI, the final concentration of 0.03% could inactivate TGEV at 12 h, and final concentrations of 0.04% and 0.05% could inactivate TGEV at 6 h. Therefore, we selected the final concentration of 0.2% FA with 24 h inactivation of TGEV, the final concentration of 0.01% BPL with 18 h inactivation of TGEV, and the final concentration of 0.04% BEI with 12 h inactivation of TGEV for the follow-up experiments, respectively. Sterility tests were conducted in common nutrient agar and ordinary broth at 37 °C for 30 h, and no bacterial growth was observed on the culture medium.

**Table 1:** Inactivation results of TGEV with FA, BPL and BEI

Inactivating agents	Final concentration (v/v) (%)	Time length of inactivation (h)						Temperature of inactivation (℃)
		6	12	18	24	30	36	
FA	0.1	−	−	+	+	+	+	37
	0.2	−	−	+	+	+	+	
	0.3	−	−	+	+	+	+	
BPL	0.01	−	+	+	+	+	+	4
	0.02	−	+	+	+	+	+	
	0.03	+	+	+	+	+	+	
BEI	0.03	−	+	+	+	+	+	30
	0.04	+	+	+	+	+	+	
	0.05	+	+	+	+	+	+	

− The TGEV was still alive with infectivity; + the TGEV was inactivated thoroughly

### Detection of TGEV-specific IgG by ELISA

**Fig. 1:**
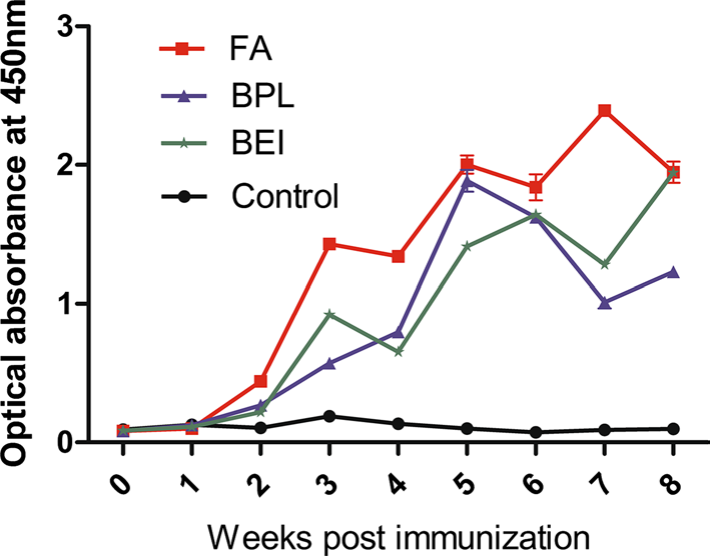
Detection of TGEV-specific IgG in mice sera. Mice sera were collected weekly after the first immunization and TGEV-specific IgG was detected by ELISA kit (n = 5). Bars represent the mean (± standard deviation) of three replicates per treatment in one experiment

TGEV-specific IgG antibody in serum was detected by ELISA in each group at weekly intervals. As shown in Fig. 1, IgG antibody was produced in FA group, BPL group and BEI group after immunization when compared with the PBS control group. The TGEV-specific IgG antibody increased significantly after three weeks of the first immunization. The level of TGEV-specific IgG antibody in FA group peaked at 49 dpi. In BPL group, the IgG antibody titer reached the highest level at 35 dpi. In BEI group, IgG antibody titer peaked at 56 dpi. Above all, the FA group produced earlier and stronger IgG than that of BEI and BPL groups, while the BEI group could produce much longer-term IgG than that of FA and BPL groups.

### Analysis of CD4^+^, CD8^+^T lymphocytes

**Fig. 2:**
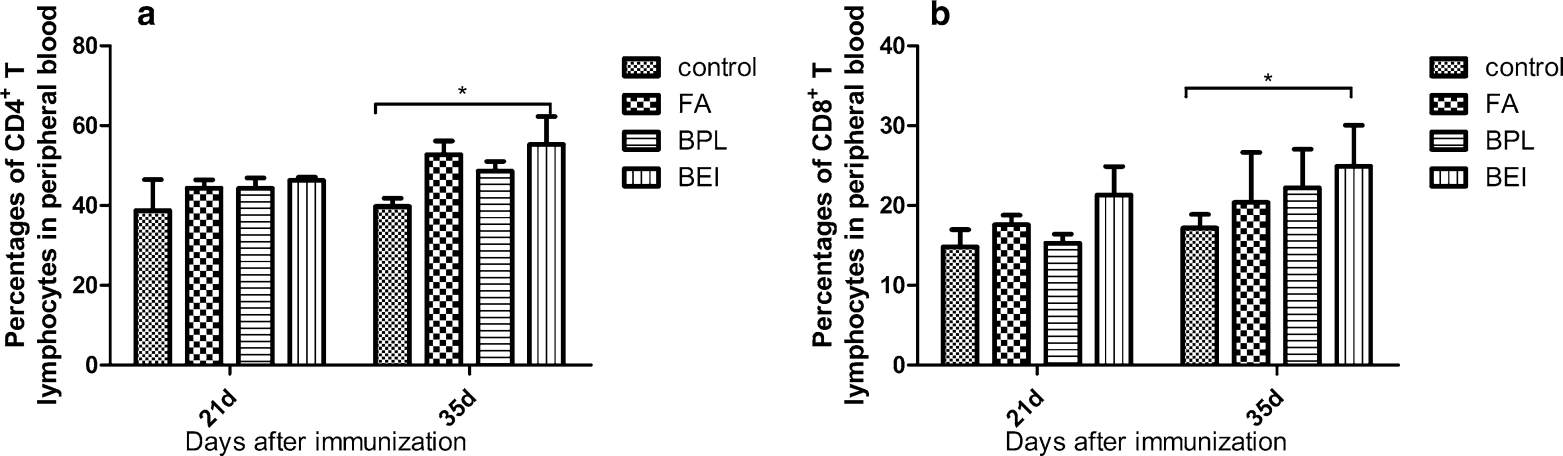
The positive rates of CD4^+^ and CD8^+^ T lymphocyte subsets were analyzed by flow cytometry. At 21 day post-inoculation (dpi) and 35 dpi, blood samples were collected from mice (n = 3). **a** The positive rates of CD4^+^ T lymphocyte subset. **b** The positive rates of CD8^+^ T lymphocyte subset. Bars represent the mean (± standard deviation) of three replicates per treatment in one experiment. Statistical significance was indicated by *P < 0.05 (significant) compared with control group

At 21 dpi and 35 dpi of the first immunization, blood samples were randomly collected from mice in each group, respectively. The positive rates of CD4^+^, CD8^+^ T lymphocyte subsets were analyzed by flow cytometry (Fig. 2). The results showed that the positive rates of CD4^+^ T lymphocyte subsets in FA group, BPL group and BEI group were 44.4 ± 2.902%, 44.3 ± 3.661% and 46.3 ± 1.178% at 21 dpi, respectively, slightly higher than that in the control group (*P* > 0.05). At 35 dpi, the positive rate of CD4^+^ T lymphocyte subset in BEI group was 55.3 ± 9.874% and reached the highest level (*P < 0.05) (Fig. 2a). The positive rates of CD8^+^ T lymphocyte subsets in FA group, BPL group and BEI group were also higher than that of the control group, and the BEI group reached the highest compared to other groups (Fig. 2b). At 21 dpi, the positive rate of CD8^+^ T lymphocyte subset was 21.3 ± 5.084% in BEI group. And at 35 dpi, the value was 24.93 ± 7.239% in BEI group, which had a significant difference compare with the control group (*P < 0.05).

### Analysis of CD4^+^IFN-γ^+^, CD4^+^IL-4^+^ T lymphocytes

**Fig. 3:**
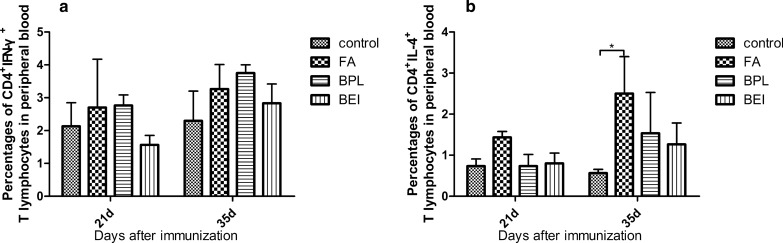
The positive rates of CD4^+^IFN-γ^+^ and CD4^+^IL-4^+^ T lymphocyte subsets analyzed by flow cytometry. At 21 dpi and 35 dpi, blood samples were collected from mice (n = 3). **a** The positive rates of CD4^+^IFN-γ^+^ T lymphocyte subset. **b** The positive rates of CD4^+^IL-4^+^ T lymphocyte subset. Bars represent the mean (± standard deviation) of three replicates per treatment in one experiment. Statistical significance was indicated by *P < 0.05 (significant) compared with control group

At 21 and 35 dpi of the first immunization, blood samples were randomly collected from mice in each group, respectively. The positive rates of CD4^+^IFN-γ^+^ T lymphocyte subsets were higher in FA group and BPL group at 21 dpi, while the BEI group was a little lower than that of the control group (Fig. 3a). At 35 dpi, all experiment groups were higher than the control group. Moreover, the positive rates of CD4^+^IFN-γ^+^ T lymphocyte subsets of 21 dpi and 35 dpi in BPL group were higher than those in other groups, with the percentages of 2.77 ± 0.45% and 3.75 ± 0.25%, respectively. For CD4^+^IL-4^+^ T lymphocyte subsets (Fig. 3b), the FA group was the highest group with the percentage of 2.5 ± 1.406%, and had a significant difference with the control group at 35 dpi (*P < 0.05).

### Result of spleen lymphocyte proliferation

**Fig. 4:**
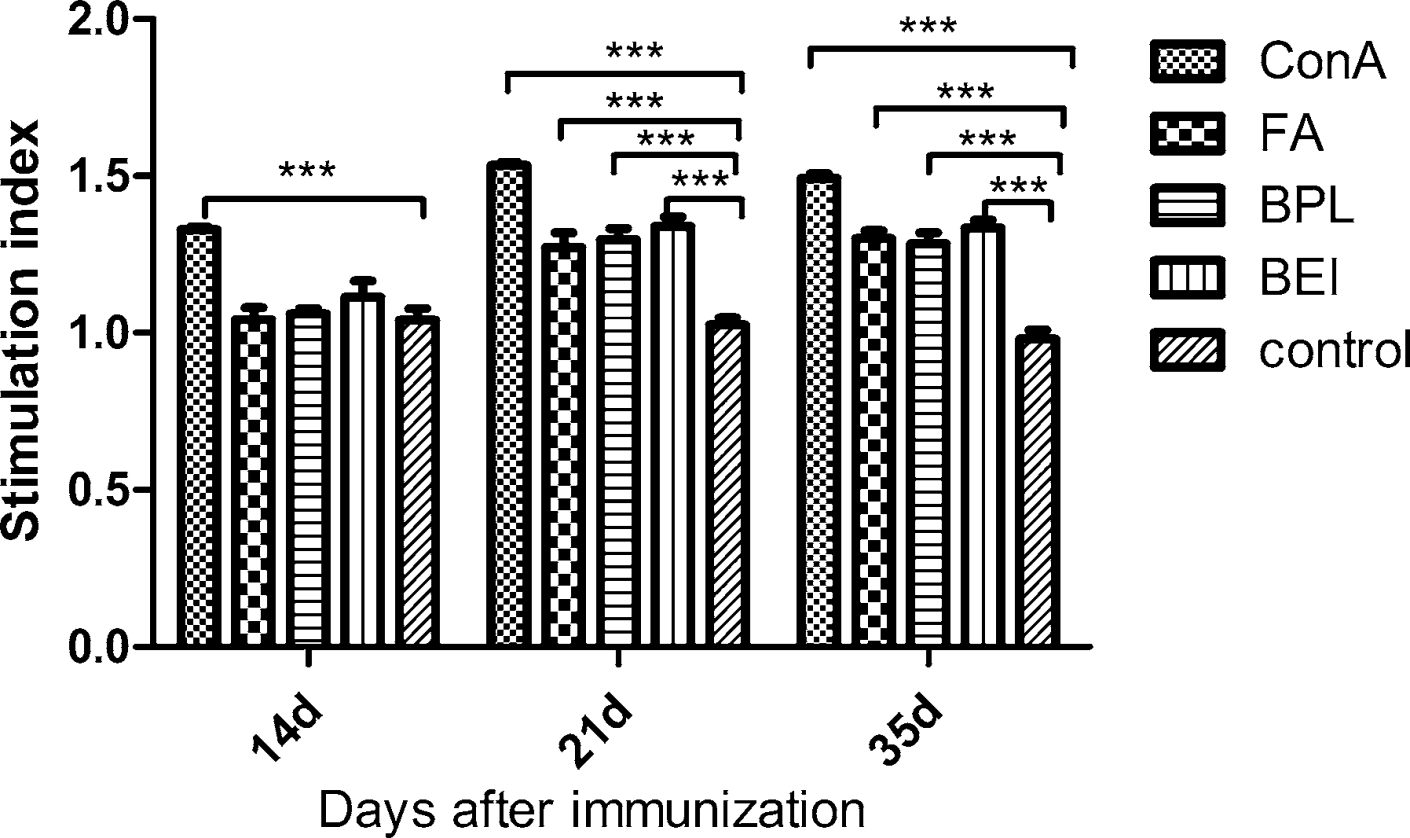
The proliferation result of spleen lymphocyte by MTT assay. Spleens of three mice in each group were collected at 14, 21 and 35 dpi, respectively (n = 3). Lymphocytes were obtained and stimulated with inactivated TGEV antigen at 37 °C for 24 h. Con A was used as the positive control, and the DMEM was used as the negative control. Bars represent the mean (± standard deviation) of three replicates per treatment in one experiment. Statistical significance was indicated by ***P < 0.001(extremely significant) compared with the negative control group

Spleens of three mice in each group were collected at 14, 21 and 35 dpi of the first immunization, respectively. The effects of spleen lymphocyte proliferation were analyzed by MTT assay and the data was shown in Fig. 4. The results indicated that at 14 dpi, there was no significant difference between the three experimental groups stimulated with inactivated TGEV antigen and the control group. At 21 dpi and 35 dpi, the stimulating effects of FA group, BPL group and BEI group were significantly enhanced compared with the negative control group (***P < 0.001). During the whole immune process, the SI values of the BEI group were higher than that of other two experimental groups, indicated that the BEI group had a stronger inducibility of spleen lymphocyte proliferation.

### Macroscopic and histopathological examination of the vaccinated mice

Three mice were randomly selected in each group and euthanized at 35 dpi for macroscopic and histopathological examination. The macroscopic examination results showed that the collected tissues, including heart, liver, spleen, lung, kidney, small intestines (jejunum and ileum) and back muscles of the injection site with vaccine had no obvious change comparing with the control group. These tissues were also investigated through H.E staining assay, and no obvious pathological changes were found both in the vaccinated mice and the control group.

## Discussion

TGEV targets the villous and crypt enterocytes of the small intestine and causes severe watery diarrhea and results in high mortality in pigs less than two weeks of age [17]. The administration of vaccines is an important way for preventing and controlling the diseases in animals. In recent years, TGEV had decreased in many countries of the world. However, TGEV is still outbreak in swine farms in some Asia countries such as China and Korea [18, 19]. At present, different types of vaccines are available. DNA vaccines of TGEV were constructed and showed good humoral, mucosal, and cellular immunogenicity in piglets [5, 20–22]. Inactivated TGEV vaccines with adjuvants of CpG DNA or nano silicon enhanced the humoral and cellular immune responses [23]. Among these vaccines, commercial vaccines, including inactivated and attenuated vaccines, have been widely used in China, and the inactivated vaccine is the better choice for its excellent safety and has been given higher priority in pig industry.

Inactivation procedures should not affect the immunogenicity of the viral antigen. In vitro potency of FA inactivated NDV vaccine was lower than that of BPL inactivated NDV [11]. Inactivated influenza vaccine with BPL has resulted in undetectable infectivity levels, while FA treated virus retained very low infectious titers. BPL inactivated influenza virus induced higher levels of TLR7 activation than that of FA inactivated virus [12]. Since the action mode of FA, BPL and BEI is different, in this study, the three inactivating agents were prepared to inactivate the TGEV HN-2012 strain, and the effects of the three inactivating agents were assessed by TGEV-specific IgG, the positive rates of CD4^+^, CD8^+^ T lymphocyte subsets, CD4^+^IFN-γ^+^, CD4^+^IL-4^+^ T lymphocyte subsets, lymphocyte proliferation and the histopathological examination. Inactivation protocols were optimized by different concentrations at different times of FA, BPL and BEI, and the immunogenicity and safety of the vaccines were tested in mice. The commercial inactivated vaccines of TGEV were mainly inactivated by FA, and for the purpose to compare the three inactivated agents, we did not set commercial inactivated TGEV vaccines as a control group.

It is clear that the level of IgG antibody is an important indicator to evaluate the effect of the vaccine. In our study, three experimental groups induced specific TGEV-IgG antibodies in mice after immunization. These results showed that the TGEV inactivated vaccines induced humoral immunity effectively and efficiently. The specific IgG antibody induced by the vaccines were higher than those in the control group at 14 dpi (*P* > 0.05). At 21 dpi, the differences were statistically significant compared with the control group (***P < 0.001), and the IgG levels of TGEV in three experimental groups increased between 14–56 dpi. In addition, the values of the FA group peaked at 49 dpi and decreased thereafter, the values of the BPL group peaked at 35 dpi and then decreased, and the values of the BEI group peaked at 56 dpi. These results showed that the FA and BEI groups were better in eliciting humoral immune response to TGEV than the BPL group.

Inactivated vaccine can induce T lymphocyte proliferation, and the change of T lymphocyte ratios could reflect the state of cellular immune response. In this study, spleen-derived lymphocytes from immunized mice showed that vaccine immunization significantly induced T cell proliferation. T lymphocyte proliferation induced by BEI group was higher than other two experimental groups, but no significant differences (*P* > 0.05) were observed among them. At 21 dpi and 35 dpi, the SI values of three experimental groups were significant higher than that of the control group (*P < 0.05). The results confirmed that the experimental groups could induce cellular responses.

T lymphocytes are important effector cells for protection against virus infection. Previous studies showed that T cells have protective effects in animals [24, 25]. CD4^+^, CD8^+^ T lymphocyte subsets are critical to the production of immunity to virus, which were analyzed in this study. The numbers of CD4^+^ and CD8^+^ T lymphocytes increased in the peripheral blood of mice from experimental groups, and higher at 35 dpi than that of 21 dpi. The BEI group was the highest group with the significant difference (*P < 0.05) compared to the control group. In addition, these results were consistent with the BEI group inducing TGEV-specific IgG levels and T lymphocyte proliferation higher than the other two groups.

Th cells are differentiated into Th1 and Th2 lymphocyte subsets. Th1 cells play an important role in regulating cellular immune responses and mostly influenced by IFN-γ, while Th2 cells activate the humoral and mucosal immunities that mainly controlled by IL-4 [26, 27]. In this study, we detected higher levels of CD4^+^IFN-γ^+^, CD4^+^IL-4^+^ T lymphocyte subsets in TGEV vaccine groups, and the results indicated that inactivated TGEV vaccine could activate Th1 and Th2 immune responses. The higher positive rate of CD4^+^IFN-γ^+^ T lymphocyte subset was observed in the BPL group at 35 dpi, and the higher positive rate of CD4^+^IL-4^+^ T lymphocyte subset was observed in the FA group at 35 dpi. However, the differences among the three experimental groups were not significant (*P* > 0.05). The differences in the potencies of FA, BPL and BEI inactivated vaccines may be related to the fact that protein is the primary target of FA, nucleic acid is the mainly target of BEI, while both protein and nucleic acid are attacked by BPL [28, 29].

In this study, the whole virus of TGEV was used as the viral antigen which contained all the proteins of TGEV. TGEV could induce FcRn expression via the NF-kB pathway in IPEC-J2 cells, and TGEV N protein and TGF-β up-regulated FcRn expression [30]. Moreover, studies have shown that the use of fusion proteins of the Fc fragment as immunogenic antigens can improve the efficacy of vaccines [31, 32]. Major problems have been reported in the development of vaccines for severe acute respiratory syndrome (SARS) caused by a coronavirus, in which live virus uptake mediated by Fc receptors may have enhanced viral infection [33]. Thus, it is worth investigating that it may be necessary to bias the immune response towards neutralizing the epitopes on S protein in the future.

## Conclusions

In summary, to our knowledge, this is the first study to evaluate the effects of three inactivating agents on the immunogenicity of inactivated TGEV vaccine. Our results showed that the FA group had better effects on humoral immunity, while the BEI group showed its excellent effect on cellular immunity. Considering the effects of both humoral and cellular immunities, BEI might be the better inactivating agent to TGEV than FA and BPL.

## Data Availability

All data generated or analyzed during this study are included in this published article.

## Abbreviations

TGEV: Transmissible gastroenteritis virus
FA: Formaldehyde
BPL: β-Propiolactone
BEI: Binary-ethylenimine
PEDV: Porcine epidemic diarrhea virus
NDV: Newcastle disease virus
ST: Swine testis
DMEM: Dulbecco’s modified eagle medium
FBS: Fetal bovine serum
P: Passage
CPE: Cytopathic effect
BEA: 2-Bromo-ethylamine HBr
ELISA: Enzyme-linked immunosorbent assay
OD: Optical density
dpi: Day post-inoculation
BV510: Brilliant Violet 510
FITC: Fluorescein isothiocyanate
APC: Allophycocyanin
PE: Phycoerythrin
ConA: Concanavalin A
MTT: Methylthiazoltetrazolium
DMSO: Dimethyl sulfoxide
SI: Stimulation index
SD: Standard deviation
